# Differences in physicochemical properties of pectin extracted from pomelo peel with different extraction techniques

**DOI:** 10.1038/s41598-024-59760-7

**Published:** 2024-04-22

**Authors:** Yangyang Yu, Ping Lu, Yongfeng Yang, Huifu Ji, Hang Zhou, Siyuan Chen, Yao Qiu, Hongli Chen

**Affiliations:** 1 College of Tobacco Science, Henan Agricultural University, Zhengzhou, 450002 China; 2 China Tobacco Fujian Industrial Co., Ltd, Xiamen, 361012 China; 3 China Tobacco Henan Industrial Co., Ltd, Zhengzhou, 450000 China

**Keywords:** Pomelo peel, Pectin yield, Apparent viscosity, Antioxidant activity, Biochemistry, Biological techniques

## Abstract

In order to obtain high yield pomelo peel pectin with better physicochemical properties, four pectin extraction methods, including hot acid extraction (HAE), microwave-assisted extraction (MAE), ultrasound-assisted extraction, and enzymatic assisted extraction (EAE) were compared. MAE led to the highest pectin yield (20.43%), and the lowest pectin recovery was found for EAE (11.94%). The physicochemical properties of pomelo peel pectin obtained by different methods were also significantly different. Pectin samples obtained by MAE had the highest methoxyl content (8.35%), galacturonic acid content (71.36%), and showed a higher apparent viscosity, thermal and emulsion stability. The pectin extracted by EAE showed the highest total phenolic content (12.86%) and lowest particle size (843.69 nm), showing higher DPPH and ABTS scavenging activities than other extract methods. The pectin extracted by HAE had the highest particle size (966.12 nm) and degree of esterification (55.67%). However, Fourier-transform infrared spectroscopy showed that no significant difference occurred among the different methods in the chemical structure of the extracted pectin. This study provides a theoretical basis for the industrial production of pomelo peel pectin.

## Introduction

Pectin is a macromolecular polysaccharide, which was composed of backbone of α-1,4-galacturonic acids^1^. Pectin is widely used in food, cosmetics, and pharmaceutical industries because of its gelling, thickening, stabilizing, and rheological properties^2^. It also provides many health benefits for humans, such as lowering cholesterol and blood sugar, inhibiting cancer cell growth, and improving the immune system^3^. Physicochemical properties of pectin depends on its structure, which is considerably influenced by the extraction methods^4^. Therefore, it was imperative to investigate how different extraction methods affect the physicochemical properties of extracted pectin.

Hot acid extraction (HAE), a conventional extractive method, is the most preferred method in terms of cost and efficiency in practical manufacturing^5^. However, large quantities of effluent are produced during HAE, causing environmental pollution concerns. Additionally, the method is time-consuming and limits pectin yield. Recently, some highly efficient pectin extraction technologies have been adopted, such as ultrasound-assisted extraction (UAE)^6^, microwave-assisted extraction (MAE)^7^, and enzymatic assisted extraction (EAE)^8^. MAE is a green method whereby microwave energy, and microwave energy is considered a suitable source of extraction energy as it does not break chemical bonds in a compound^9^. UAE requires relatively short time and low amount of solvent, and considered more environmentally-friendly^10^. Pectin extraction by MAE and UAE have many advantages, such as reducing wastewater production, increasing yield and purity of extracted pectin^11^. It was reported that the pectin extracted by EAE contained higher amount of arabinose than HAE, and showed better immunomodulatory properties^12^. However, the physicochemical properties of pectin extracted through the different methods varied considerably. Dranca et al.^13^ investigated the effects of HAE, UAE, MAE, and EAE on the physicochemical properties of apple pectin. The results showed that the pectin obtained by UAE and MAE contained higher galacturonic acid content and higher viscous solutions than those of HAE and EAE.

Pomelo (*Citrus grandis* (L.) Osbeck) is a member of the citrus family. Pomelo peel comprises approximately 40% of the fruit's weight and contains natural chemical components, such as cellulose, flavonoids, and essential oil. Pomelo peel is a potential source of pectin^14^. However, pomelo peel has not been exploited as a pectin resource and is usually discarded as garbage. Only a few studies have investigated the physicochemical properties of pomelo pectin, and the optimal extraction method of pomelo pectin has not been considered. Besides, it is clear that the influences of different extracted methods on physicochemical properties of pomelo peel pectin. The structure of pectin depends on the plant source and the method of extraction, which is a determinant factor on its physicochemical properties and application^15^. Therefore, this study compared HAE, MAE, UAE, and EAE in terms of obtained yield and physiochemical properties in pomelo peel. The comparisons of results are helpful to associations of physiochemical properties and extraction methods, and offer a basis in the industrial production of pomelo peel pectin.

## Materials and methods

### Materials

Pomelo fruits of the same batch and ripeness were obtained from Guangzhou, China. The peels were cut and separated from the flesh. Subsequently, the peels were dried in an oven at 60 °C until no change was observed in the weight. The dried peels were ground and sieved (250–300 μm) to obtain pomelo peel powder. All reagents and chemicals used in this study were of analytical grade and supplied by Sigma-Aldrich, St. Louis, Missouri, USA.

### Pectin extraction

1. Hot acid extraction (HAE): HAE of pectin was carried out based on the method of Torralbo et al.^16^ with minor modification. Pomelo peel powder (10 g) was mixed with 0.1 M water–citric acid solution (100 mL; 2.0 pH), and then the mixture was kept in a water bath at 90 °C with continual agitation for 60 min.

After each extraction, the mixture was centrifuged (5000 g, 10 min), and the supernatant was collected. Then, the supernatant was precipitated by adding ethyl alcohol (2:1, v/v) and kept at 4 °C for 12 h. The precipitated pectin was collected, and all the extractions were performed in triplicate. Finally, the pectin was freeze dried and stored at ambient conditions for further analyses.

(2) Microwave-assisted extraction (MAE): MAE of pectin was carried out based on the method described by Li et al.^17^. Pomelo peel powder (10 g) was mixed with 0.1 M water–citric acid solution (100 mL; 2.0 pH) in a beaker. The mixing was performed in an experimental microwave oven (power of 50 W, frequency of 2450 MHz, PreeKem Scientific Instruments Co., Ltd. China) at 100 °C for 4 min. The other steps were kept the same as that of HAE.
(3) Ultrasound-assisted extraction (UAE): UAE of pectin was carried out based on the method described by Wang et al.^18^ with slight modification. Pomelo peel powder (10 g) was mixed with 0.1 M water–citric acid solution (100 mL; 2.0 pH). The mixture was sonicated for 20 min by an ultrasonic device (ultrasound intensity, 480.2 W/cm^2^; temperature, 45 °C) and stirred continuously. The other steps were kept the same as that of HAE.
(4) Enzyme-assisted extraction (EAE): Pomelo peel powder (10 g) was mixed with water–citric acid solution (100 mL; 4.5 pH). Then, the extraction process began by adding 2 g of cellulase enzyme (5000 U/g, food grade, Imperial Jade Bio-technology Co., Ltd.) to the mixture, followed by conducting the extraction at 47 °C for 10 h with constant shaking at 200 rpm. After the extraction, the samples were heated at 90 °C for 5 min to inactivate the enzyme. Afterward, the samples were cooled^19^. All other steps remained the same as the HAE.

### Extraction yield and particle size

The extraction yields (EY, %) of the pectin obtained were calculated by the Eq. (1):

1 $${\text{EY}} \left( \% \right) = \frac{weight\;of\;dried\;pectin \left( g \right)}{{weight\;of\;pomelo\;peel\;powder \left( g \right)}} \times 100\% ,$$

The particle size of the pectin was measured using particle size analyser (Malvern, UK) based on a previous report^20^.

### Chemical composition analysis

Protein content was determined by the Bradford method with bovine serum albumin as the standard^21^. The total phenolic content was calculated using the Folin-Ciocalteu method as described by Hosseini et al.^22^. The methoxyl content of the pectin was calculated based on the previous report^23^, The galacturonic acid content was measured with the m-hydroxydiphenyl method described by Dranca et al.^13^. The degree of esterification (DE) of the pectin sample was measured based on a previous study^24^.

### Fourier-transform infrared spectroscopy (FTIR)

Pectin samples were mixed with KBr (1:100) and pressed into pellets before the measurement. FTIR spectra was determined using a Nicolet 5700 FTIR spectrometer (Thermo Fisher Scientific, USA) in the wavenumber range of 400–4000 cm^−1^ at a resolution of 4 cm^−1^.

### Thermal analysis

Thermal analysis of pectin samples was performed with differential scanning calorimetry (DSC) (DSC 8500, PerkinElmer, USA). The pectin sample (5 mg) was sealed in an aluminum pan with a pinhole, and heated form 30 to 300 °C (10 °C/min).

### Emulsifying activity (EA) and emulsion stability (ES)

The EA and ES of the pectin samples were measured based on the method described by Zhao et al.^25^. Emulsions were prepared by mixing 5 mL corn oil and 5 mL pectin solutions (0.5%, w/w), and the mixture was centrifuged (5000×*g* for 5 min). After 24 h at 24 °C, the volume of the emulsified layer was measured. The EA and ES were calculated by the Eqs. (2) and (3), respectively.

2 $$EA \left( \% \right) = \frac{{the\;volume\;of\;the\;emulsified\;layer \left( {{\text{mL}}} \right)}}{{the\;whole\;volume\;of\;the\;mixture \left( {{\text{mL}}} \right)}} \times 100\% ,$$

3 $$ES \left( \% \right) = \frac{{the\;volume\;of\;the\;remaining\;emulsified\;layer\;after\;30\;days \left( {{\text{mL}}} \right)}}{{the\;volume\;of\;the\;emulsified\;layer \left( {{\text{mL}}} \right)}} \times 100\% ,$$

### Rheological properties

The rheological property was investigated with AR1500ex Rheometer (TA Instruments, New Castle, USA) with a 50 mm parallel plate according to a previous study. The pectin sample 1% (w/w) was dissolved in distilled water. The Rheological properties analysis of pectin was conducted under a shear rate from.

### Antioxidant capacity

DPPH radical scavenging activity was measured based on a previous report^26^. Briefly, the sample (2.5 mL) was mixed with 1.5 mL DPPH solution (100 mM). After 20 min, the absorbance was measured at a wavelength 517 nm using a spectrophotometer. The DPPH radical scavenging activity was calculated by the Eq. (4):

4 $$DPPH\;radical\;scavenging\;activity \left( \% \right) = \frac{{\left( {A_{0} - A_{n} } \right)}}{{A_{0} }} \times 100\% ,$$

where ${A}_{0}$ and ${A}_{n}$ are the absorbance values of the control and test samples, respectively.

ABTS radical scavenging activity was measured using a kit (Nanjing Jiancheng Bioengineering Institute Co., Ltd.).

### Data analysis

All assays and tests were performed in triplicate, and the results were expressed as means ± standard deviation. The significance of the difference was analyzed by Duncan’s multiple range test with SPSS (Version 17.0). Graphs were generated using OriginPro 2022b.

## Results and discussion

### Extraction yield and particle size by different methods

Table 1 shows that MAE (20.43%) and UAE (17.21%) achieved higher extraction yields of pectin from pomelo peel. The results were significantly (*p* < 0.05) higher than those of pectin extracted by HAE (15.36%). This result is in agreement with the results obtained by Jiang et al. on pectin extraction from seed watermelon peel^27^. This phenomenon may be due to microwaves weakening the cell wall structure and causing parenchymal cells to cleave, increasing the contact between the solvent and the extracting substance^28^. However, the extraction yield of EAE (11.94%) was the lowest, which was significantly lower than that of HAE (*p* < 0.05). Similarly, Bagherian et al.^11^ reported that MAE (27.81%) resulted in higher pectin extraction yield from grapefruit than that of HAE (19.16%). The higher extraction yield of MAE can be attributed to microwave radiation, which causes the plant tissue rupture and cell wall matrix loosening^29^. Contrary to the results of previous studies^30^, EAE did not produce a pectin yield higher than that of HAE. This result may be primarily because available enzymes cannot wholly hydrolyze plant cell walls, and enzymic response depends on some factors (e.g., reaction time, type and concentration of enzyme, temperature)^31^. Additionally, the solid-to-liquid ratio was the same (1:10) for HAE, UAE, MAE, and EAE. However, the extraction time was considerably shorter for MAE (4 min) and UAE (20 min) than for HAE (60 min).

**Table 1:** Yield and composition of pectin extracted using different methods.

	HAE	MAE	UAE	EAE
EY (%)	15.36 ± 0.84 c	20.43 ± 1.25 a	17.21 ± 0.77 b	11.94 ± 1.32 d
PS (nm)	966.12 ± 20.2 a	881.31 ± 21.61 bc	918.75 ± 20.13 b	843.69 ± 19.89 c
PC (%)	10.34 ± 0.36 b	9.06 ± 0.28 c	9.32 ± 0.24 c	12.86 ± 0.42 a
TPC (%)	1.86 ± 0.28 b	1.48 ± 0.24 b	2.42 ± 0.23 ab	2.68 ± 0.19 a
MeO (%)	7.43 ± 0.34 ab	8.35 ± 0.42 a	7.08 ± 0. 28 b	6.62 ± 0.37 c
GalA (%)	67.36 ± 0.95 b	71.36 ± 1.19 a	69.62 ± 1.74 ab	56.34 ± 1.58 c
DE (%)	55.67 ± 1.35 a	53.34 ± 1.90 ab	51.42 ± 1.63 bc	47.71 ± 1.18 d

^a^EY, extraction yield; PS, particle size; PC, protein content; TPC, total phenolic content; MeO, methoxyl Content; GalA, galacturonic acid content; DE, degree of esterification.

^b^Different letters (a, b, c, d) suggest significantly different (*p* < 0.05).

The pectin extracted by MAE (881.31 nm), UAE (918.75 nm), and EAE (843.69 nm) had a relative lower particle size than that extracted by HAE (966.12 nm) (Table 1). The effects of HAE, MAE, and UAE on pectin particle size were similar to those previously reported^32^ from the comparison of pectin extracted from black carrot pomace using HAE, MAE, and UAE. In another study, pectin particle size affected pectin’s emulsifying properties, which is important for pectin usage in food^33^.

### Chemical composition of pectin by different methods

As shown in Table 1, there was a significant difference (*p* ˂ 0.05) in the protein content of the pectin extracted using different methods. The highest protein content was found in the pectin extracted by EAE (12.86%). The pectin of UAE (9.32%) had a lower protein content compared to HAE (10.34%) possibly due to the hydrolysis of protein caused by ultrasound and papain, leading to a decrease in protein content^30^. Although the pectin content of the MAE (9.06%) extraction method was lower compared to other methods, this disparity may be attributed to the covalent bond formed between the amino group on the protein's side chain and the terminal hydroxyl groups in the polysaccharides via the Maillard reaction^34^.

Compared with HAE, EAE and UAE displayed higher total phenolic content in pectin (Table 1). The cell wall's structural integrity may be weakened by enzymes and ultrasonic methods, leading to the release of polyphenols from pomelo peel. This occurs while minimizing or reducing the structural alterations of polyphenols at lower temperatures. Whereas, HAE and MAE needed a higher temperatures (> 80 °C), avoiding or decreasing structural changes of polyphenols and decreased the total phenolic content in pectin^13^. This result was consistent with previous results reported by Bai et al.^28^ that the potato polysaccharide obtained by EAE or UAE has higher total phenolic content.

The gel capacity of pectin is significantly influenced by the methoxyl content. As shown in Table 1, the methoxyl content of pectin obtained by MAE (8.35%) was significantly higher (*p* < 0.05) than other extraction methods, which is similar to the result of pectin extracted from kinnow peel^23^. The elevated methoxyl concentration in pectin obtained via MAE could be ascribed to the existence of an esterified carboxyl group^35^. According to the report, pectin with a higher methoxyl content is more soluble in water than pectin with a lower methoxyl content^36^. The lowest methoxyl content was observed in the pectin extracted from EAE (6.86%). The methoxyl content of UAE and HAE was 7.43% and 7.28%, which is similar to the result of pectin extracted from lime peel^37^. The methoxyl content of pectin was not significant difference among the UAE, EAE and HAE. Commercial pectin typically contains 8–11% methoxyl content and can form high sugar gels exceeding 65%. In contrast, the presence of low methoxyl pectin allows for the generation of gels containing sugar levels below 7.0%^36^.

The galacturonic acid is regarded as the backbone of the pectin molecule, and the content of galacturonic acid could determine the purity of pectin^18^. Pectin extracted by MAE (71.36%) and UAE (69.62%) had a higher galacturonic acid content than that extracted by HAE (Table 1). The galacturonic acid content of pectin extracted from lime peel using MAE was higher than the HAE^34^. Similar results have been shown by the pectin extracted from sisal waste using UEA^38^. According to previous reports that MAE and UAE have a superior capability to entirely release pectic compounds with more galacturonic acid from deeper parts of the plant structure^38^. However, for EAE pectin (56.34%), the galacturonic acid content was lower than that of the HAE pectin (67.36%). Similar values were observed from the date and lemon pectin (41.5 to 74.5%) which were extracted using acidified water. The content was consistent with the extracted yield of pectin extracted by different methods. According to the FAO and EU requirements, the galacturonic acid content in industrial pectin should be at least 65%^39^. Therefore, the pectin extracted by EAE is unsuitable for forming strong gels in the food industry.

The DE had a significant impact on the functional properties of pectin. Table 1 indicates that the DE of pectin obtained through HAE (55.67%) surpassed that of pectin obtained through alternative techniques. Nevertheless, EAE achieved the lowest DE of pectin (47.71%), significantly inferior to that attained by HAE. The DE can be categorized as low methoxyl pectin due to its concentration falling below 50%^40^. Additionally, the DE of pectin extracted by MAE (53.34%) and UAE (51.42%) was lower than that extracted by HAE. These results are consistent with previous studies, showing that the DE of pectin obtained by UAE and MAE was lower than that obtained by HAE^41^. The pectin extracted through MAE and UAE is subjected to harsh conditions, which leads to higher de-esterification of polygalacturonic chains, resulting in a decreased DE^11,42^.

### FTIR

**Figure 1:**
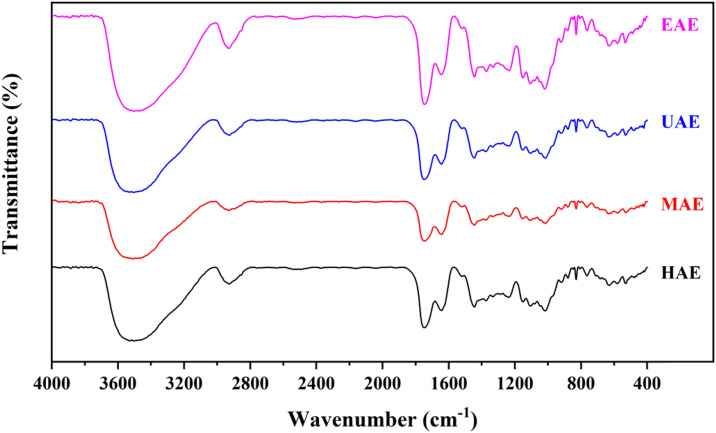
FT-IR spectra of pectin extracted using different methods.

Figure 1 illustrates the FTIR spectra of the pectin obtained by different extraction methods. The pectin extracted by the different methods exhibited similar FTIR spectra, showing that the main structure of the pectin was not influenced by the different extraction methods, further confirming that the extracts were pectin. For all the pectin samples, the wide and strong absorption at 3500 cm^−1^ was caused by the stretching of oxygen–hydrogen bond (–OH), and the absorption peak at 2936 cm^−1^ was attributed to the C–H bond (including CH, CH_2_, and CH_3_) vibration (Table 2)^43^. Meanwhile, the bonds observed in the regions from 1735 to 1750 cm^−1^ and 1600 to 1400 cm^−1^ were identified as the esterified carboxyl (–COOR) and free carboxylic groups (–COO^−^), respectively. The absorption bands at 1010 cm^−1^ indicate that the existence of pyranose. According to the FTIR spectra, a polysaccharide-rich polygalacturonic acid was the pectin extracted from the pomelo peel using different methods, and the results were in agreement with those of pectin extracted from jackfruit rags^27^. Additionally, the pectin FTIR patterns extracted with the different methods were similar.

**Table 2:** The summary of FT-IR spectra analysis.

Wavenumber (cm^−1^)	3500	2936	1735–1750	1600–1400	1010
Structural feature	–OH	C–H bond (including CH, CH2, and CH3)	Esterified carboxyl (–COOR)	Free carboxylic groups (–COO−)	Pyranose

### Thermal properties

**Figure 2:**
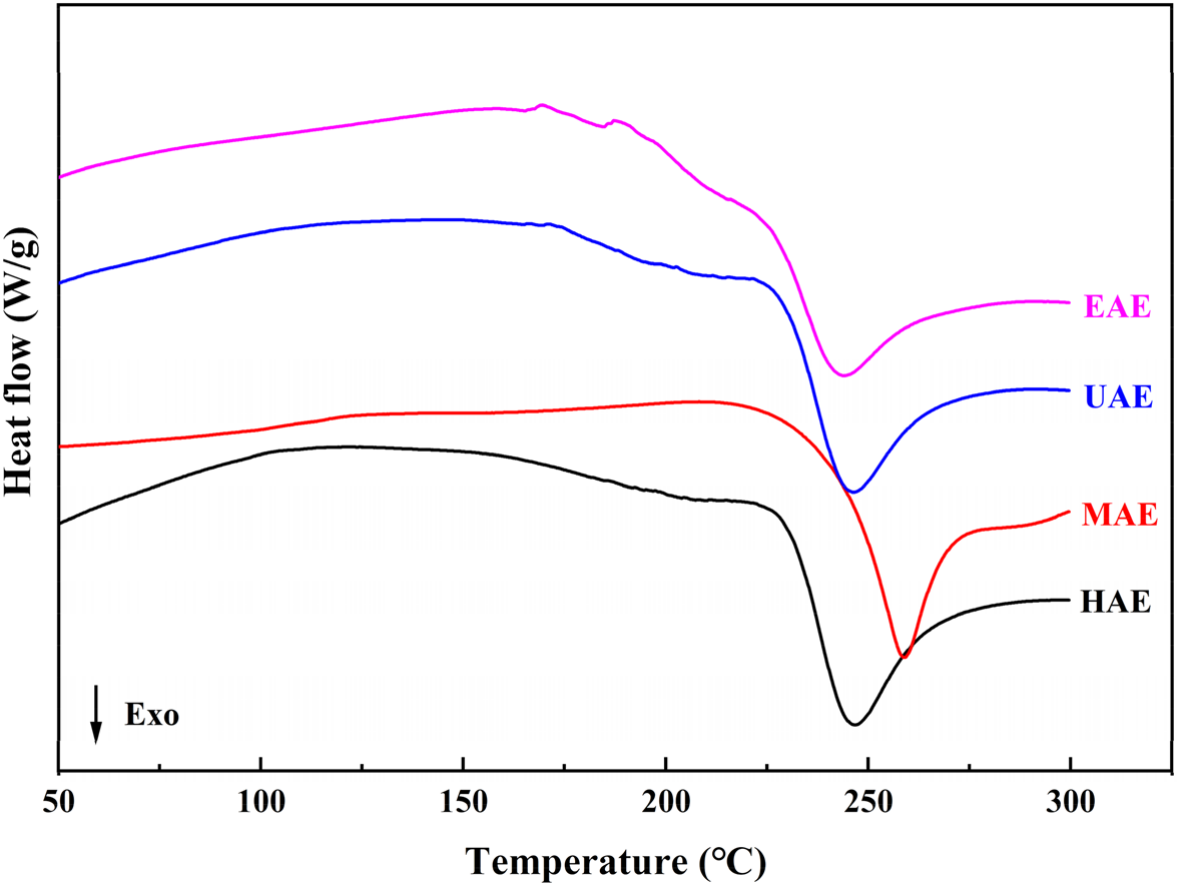
DSC thermograms of pectin extracted using different methods. (Heating rates of 10 °C min^−1^ from 50 to 300 °C).

Figure 2 shows that apparent endothermic peaks (melting temperature) did not occur. The endothermic peaks resulted from water evaporation, and our results are consistent with those of Qin et al.^25^, showing that no bound water was removed from the pectin samples. All the pectin samples showed exothermic peaks between 240 and 265 °C, where the pectin degradation began. The maximum exothermic peaks for CHE, MAE, UAE, and EAE were at 246.2, 260.1, 244.7, and 242.9 °C, respectively. The pectin obtained by MAE had a broader exothermic peak, indicating that the pectin had a higher thermal stability. Additionally, the pectin obtained by HAE had a broader exothermic peak than that of other methods, indicating its wider molecular weight distribution, which correlates with molecular weight distribution^13^.

### Emulsifying properties

**Figure 3:**
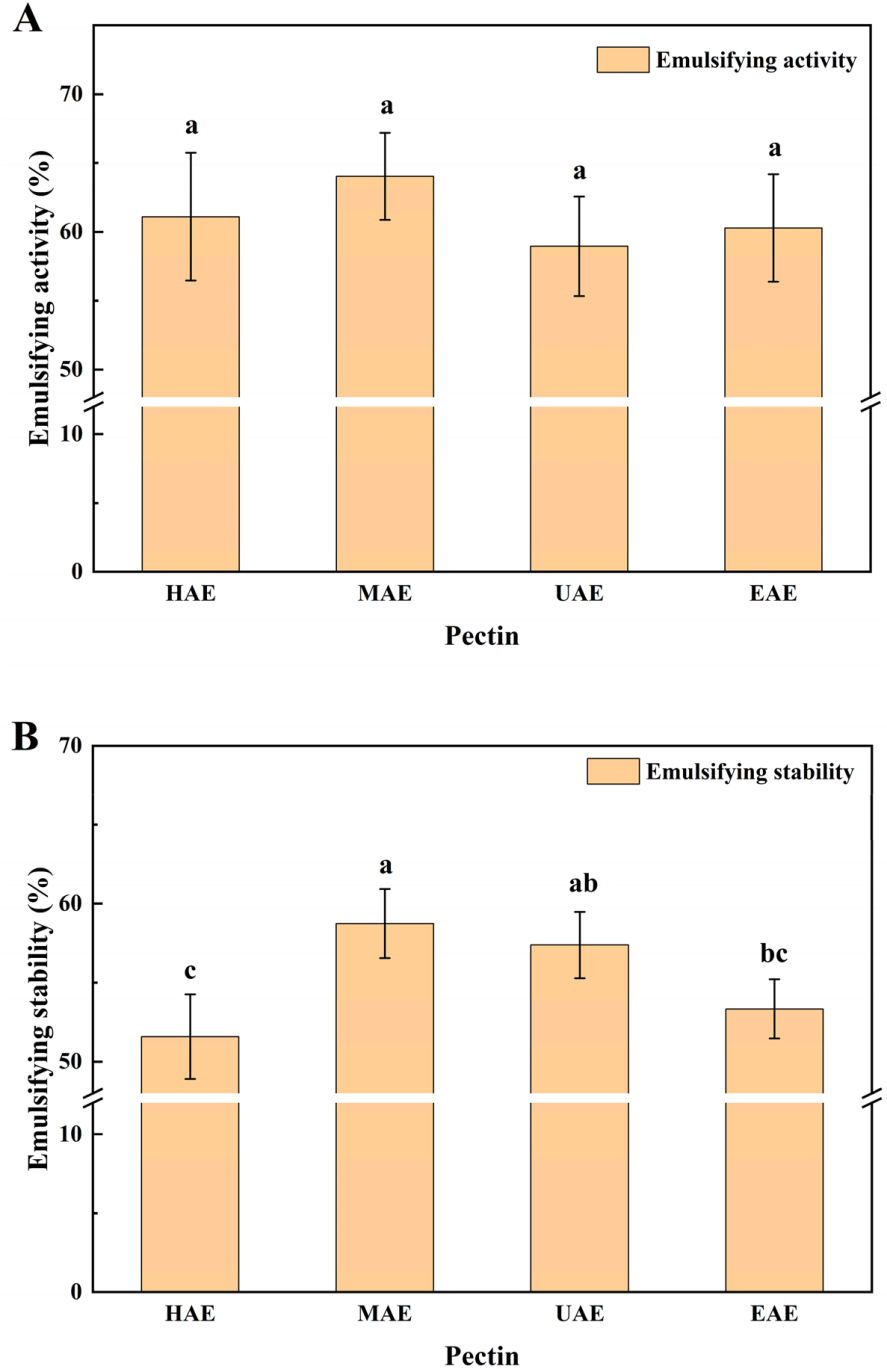
EA (**A**) and ES (**B**) of pectin extracted using different methods. Different letters (a, b, c, d) indicate significantly different means at *p* < 0.05.

**Figure 4:**
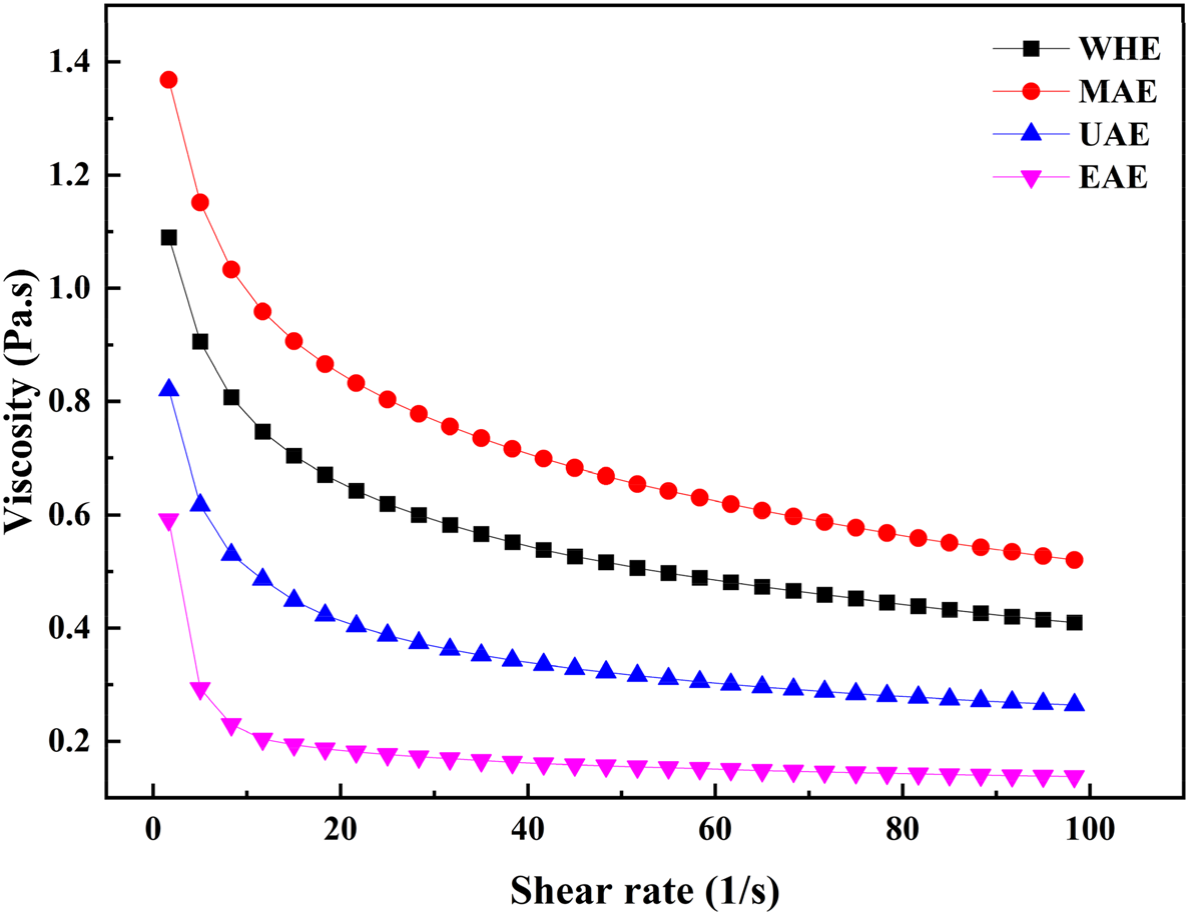
The apparent viscosity of the pectin extracted using different methods.

Pectin is utilized as a stabilizer or emulsifier in foods because of its emulsifying properties. The emulsifying properties of pectin extracted by the four methods are presented in Fig. 4, including EA and ES. The EAs of the pectin extracted by the four methods was no significant difference (*p* > 0.05) (Fig. 3A). However, the pectin extracted by HAE had the lowest ES, which was significantly lower (*p* < 0.05) than those extracted by other methods (Fig. 3B). These results are consistent with those of a previous study, showing that UAE increased the galacturonic acid content and caused increased emulsifying properties^22^, compared with those of HAE. The molecular weight, galacturonic acid content, and particle size significantly influenced the emulsifying properties of the extracted pectin^44^. In this study, the pectin extracted by UAE and MAE had higher galacturonic acid content and lower particle size, which increased the emulsifying properties.

### Rheological properties analysis of pectin

The pectin solution samples, as shown in Fig. 4, showed a decrease in viscosity and an increase in shear rate, suggesting that the fluid was pseudoplastic. The gradual weakening of pectin's intermolecular strength during the test may account for the observed shear-thinning trend across all the curves. The shear-thinning behavior is a result of the pectin's intermolecular forces decreasing as the shear rate increases^45^. Apparently, the viscosity of pectin extracted by MAE was higher than that of the other methods. This result is consistent with the findings of Chen et al.^46^, who reported that the pectin extracted by MAE had higher molecular weight and weakened relative motion of molecules, and thus showed a high apparent viscosity. However, the pectin extracted by UAE retained low and stable viscosity.

### Antioxidant activities

**Figure 5:**
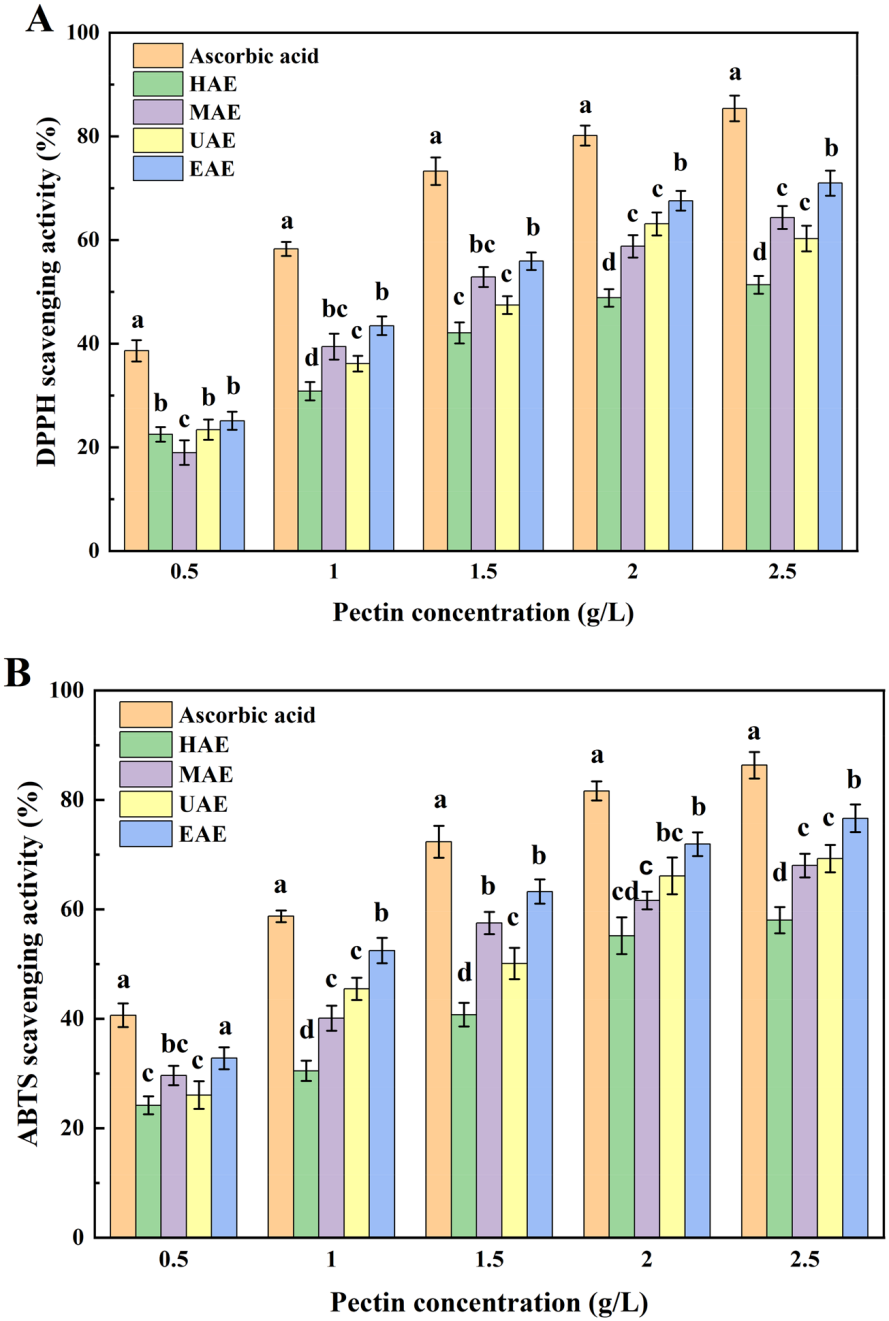
DPPH (**A**) and ABTS (**B**) radical scavenging activities of the pectin extracted by different methods at various concentrations. Different letters (a, b, c, d) indicate significantly different means at *p* < 0.05.

Pectin has antioxidant activity due to the hydroxyl group of the molecule^47^. The colour of samples changes from purple to yellow when the free radicals of DPPH are scavenged by antioxidant compounds^48^. After the reaction between ABTS• + and an antioxidant, ABTS• + is converted to its non-radical form by donating an electron^43^. Therefore, the DPPH and ABTS radical scavenging activities are widely used in measuring antioxidant activities^49,50^. As shown in Fig. 5A,B, all the pectin with different extraction showed DPPH and ABTS radical scavenging activities, showing the antioxidant activities positively correlated with their concentration from 0.5 to 2.5 mg/mL. These results are consistent with the results reported by Gharibzahedi et al.^43^. However, it is difference that the antioxidant properties of pectin obtained by different extraction methods. These results indicated a significant correlation between the antioxidant activities and extraction methods.

At same concentration, the pectin extracted by EAE had the highest DPPH and ABTS radical scavenging activities. The results are consistent with the findings of Wang et al.^1^, who reported that extracted apple pectin had considerably higher antioxidant activity than that of HAE. This phenomenon could be attributed that the pectin obtained by EAE displayed higher total phenolic content and lower particle size, which improving more reactive sites with free radicals and increased antioxidant activities^28^. Additionally, the pectin extracted by UAE and MAE had substantially higher DPPH (Fig. 5A) and ABTS (Fig. 5B) radical scavenging activity ranges than that extracted by HAE at same concentration. The higher antioxidant capacity of pectin can be attributed to the higher galacturonic acid content of pectin extracted by UAE and MAE. It was reported by Wang et al.^51^ that the antioxidant activity of pectin also depends on the amount of galacturonic acid besides total phenolic content. These findings indicate that by using the right method and parameters, it is possible to produce pectin with potent antioxidant activity.

## Conclusions

In this study, HAE, UAE, MAE, and EAE methods were used to extract pectin from pomelo peel, and their effects on pectin physicochemical properties were compared. MAE and EAE resulted in the highest (20.43%) and lowest (11.94%) pectin yield from pomelo peel compared to the other methods. Fourier-transform infrared spectroscopy showed that no significant difference occurred among the different methods in the chemical structure of the extracted pectin. However, the physicochemical properties of pomelo peel pectin obtained by different methods were also significantly different. The pectin extracted by HAE had the highest particle size (966.12 nm) and degree of esterification (55.67%). The pectin obtained by MAE had the highest methoxyl content (8.35%), galacturonic acid content (71.36%), and showed a higher apparent viscosity, thermal and emulsion stability. The pectin extracted by EAE showed the highest total phenolic content (12.86%) and lowest particle size (843.69 nm), showing higher DPPH and ABTS scavenging activities than other extract methods. Therefore, EAE gained peel pectin with high biological activity, which has good application prospects in the functional-food industries.

## Data Availability

All data generated or analyzed during this study are included in this published article.

## References

[1] Mualikrishna G, Tharanathan RN (1994). Characterization of pectic polysaccharides from pulse husks. Food Chem..

[2] Wan L (2019). Comparative study on gelling properties of low methoxyl pectin prepared by high hydrostatic pressure-assisted enzymatic, atmospheric enzymatic, and alkaline de-esterification. Carbohyd. Polym..

[3] Li D (2021). Pectin in biomedical and drug delivery applications: A review. Int. J. Biol. Macromol..

[4] Zhang C (2020). Improving viscosity and gelling properties of leaf pectin by comparing five pectin extraction methods using green tea leaf as a model material. Food Hydrocoll..

[5] Koubala BB (2008). Effect of extraction conditions on some physicochemical characteristics of pectins from “Améliorée” and “Mango” mango peels. Food Hydrocoll..

[6] Xu Y (2014). Effects of ultrasound and/or heating on the extraction of pectin from grapefruit peel. J. Food Eng..

[7] Kamal MM, Ali MR, Hossain A, Shishir MRI (2020). Optimization of microwave assisted extraction of pectin from *Dillenia indica* fruit and its preliminary characterization. J. Food Process. Pres..

[8] Marić M (2018). An overview of the traditional and innovative approaches for pectin extraction from plant food wastes and by-products: Ultrasound-, microwaves-, and enzyme-assisted extraction. Trends Food Sci. Technol..

[9] Liew SQ, Teoh WH, Yusoff R, Ngoh GC (2019). Comparisons of process intensifying methods in the extraction of pectin from pomelo peel. Chem. Eng. Process..

[10] Kumar K, Yadav AN, Kumar V, Vyas P, Dhaliwal HS (2017). Food waste: A potential bioresource for extraction of nutraceuticals and bioactive compounds. Bioresour. Bioprocess..

[11] Bagherian H, Zokaee Ashtiani F, Fouladitajar A, Mohtashamy M (2011). Comparisons between conventional, microwave- and ultrasound-assisted methods for extraction of pectin from grapefruit. Chem. Eng. Proces..

[12] Güzel M, Akpınar Ö (2019). Valorisation of fruit by-products: Production characterization of pectins from fruit peels. Food Bioprod. Process..

[13] Dranca F, Vargas M, Oroian M (2020). Physicochemical properties of pectin from Malus domestica ‘Fălticeni’ apple pomace as affected by non-conventional extraction techniques. Food Hydrocoll..

[14] Liew SQ, Ngoh GC, Yusoff R, Teoh WH (2018). Acid and deep eutectic solvent (DES) extraction of pectin from pomelo (*Citrus grandis* (L.) Osbeck) peels. Biocatal. Agric. Biotechnol..

[15] Morris VJ, Gromer A, Kirby AR, Bongaerts RJ, Gunning AP (2011). Using AFM and force spectroscopy to determine pectin structure and (bio) functionality. Food Hydrocoll..

[16] Torralbo DF, Batista KA, Di-Medeiros MCB, Fernandes KF (2012). Extraction and partial characterization of *Solanum lycocarpum* pectin. Food Hydrocoll..

[17] Li D, Jia X, Wei Z, Liu Z (2012). Box–Behnken experimental design for investigation of microwave-assisted extracted sugar beet pulp pectin. Carbohyd. Polym..

[18] Wang W (2015). Ultrasound-assisted heating extraction of pectin from grapefruit peel: Optimization and comparison with the conventional method. Food Chem..

[19] Vasco-Correa J, Zapata Zapata AD (2017). Enzymatic extraction of pectin from passion fruit peel (*Passiflora edulis* f. flavicarpa) at laboratory and bench scale. LWT Food Sci. Technol..

[20] Huang X, Li D, Wang L (2018). Effect of particle size of sugar beet pulp on the extraction and property of pectin. J. Food Eng..

[21] Bradford MM (1976). A rapid and sensitive method for the quantitation of microgram quantities of protein utilizing the principle of protein-dye binding. Anal. Biochem..

[22] Hosseini SS, Khodaiyan F, Kazemi M, Najari Z (2019). Optimization and characterization of pectin extracted from sour orange peel by ultrasound assisted method. Int. J. Biol. Macromol..

[23] Kumari M, Singh S, Chauhan AK (2023). A comparative study of the extraction of pectin from Kinnow (*Citrus reticulata*) peel using different techniques. Food Bioprocess. Technol..

[24] Liu L, Cao J, Huang J, Cai Y, Yao J (2010). Extraction of pectins with different degrees of esterification from mulberry branch bark. Bioresour. Technol..

[25] Qin XD (2019). Effect of drying pretreatment methods on structure and properties of pectins extracted from Chinese quince fruit. Int. J. Biol. Macromol..

[26] Brand-Williams W, Cuvelier ME, Berset C (1995). Use of a free radical method to evaluate antioxidant activity. LWT Food Sci. Technol..

[27] Tran NTK, Nguyen VB, Van Tran T, Nguyen TTT (2023). Microwave-assisted extraction of pectin from jackfruit rags: Optimization, physicochemical properties and antibacterial activities. Food Chem..

[28] Bai C (2023). Comparison in structural, physicochemical and functional properties of sweet potato stems and leaves polysaccharide conjugates from different technologies. Int. J. Biol. Macromol..

[29] Rahmati S, Abdullah A, Kang OL (2019). Effects of different microwave intensity on the extraction yield and physicochemical properties of pectin from dragon fruit (*Hylocereus polyrhizus*) peels. Bioactive Carbohydr. Dietary Fibre.

[30] Jeong H-S (2014). Optimization of enzymatic hydrolysis conditions for extraction of pectin from rapeseed cake (*Brassica napus* L.) using commercial enzymes. Food Chem..

[31] Yuliarti O, Goh KKT, Matia-Merino L, Mawson J, Brennan C (2015). Extraction and characterisation of pomace pectin from gold kiwifruit (*Actinidia chinensis*). Food Chem..

[32] Sucheta, Misra NN, Yadav SK (2020). Extraction of pectin from black carrot pomace using intermittent microwave, ultrasound and conventional heating: Kinetics, characterization and process economics. Food Hydrocoll..

[33] Han Y (2020). Effect of metal ions and pH on the emulsifying properties of polysaccharide conjugates prepared from low-grade green tea. Food Hydrocoll..

[34] Rodsamran P, Sothornvit R (2019). Microwave heating extraction of pectin from lime peel: Characterization and properties compared with the conventional heating method. Food Chem..

[35] Voragen AGJ, Coenen GJ, Verhoef RP, Schols HA (2009). Pectin, a versatile polysaccharide present in plant cell walls. Struct. Chem..

[36] Constenla D, Lozano JE (2003). Kinetic model of pectin demethylation. Lat. Am. Appl. Res..

[37] Rodsamran P, Sothornvit R (2019). Lime peel pectin integrated with coconut water and lime peel extract as a new bioactive film sachet to retard soybean oil oxidation. Food Hydrocoll..

[38] Yang Y, Wang Z, Hu D, Xiao K, Wu J-Y (2018). Efficient extraction of pectin from sisal waste by combined enzymatic and ultrasonic process. Food Hydrocoll..

[39] Willats WGT, Knox JP, Mikkelsen JD (2006). Pectin: New insights into an old polymer are starting to gel. Trends Food Sci. Technol..

[40] Giacomazza D, Bulone D, San Biagio PL, Marino R, Lapasin R (2018). The role of sucrose concentration in self-assembly kinetics of high methoxyl pectin. Int. J. Biol. Macromol..

[41] Guandalini BBV, Rodrigues NP, Marczak LDF (2019). Sequential extraction of phenolics and pectin from mango peel assisted by ultrasound. Food Res Int..

[42] Wai WW, Alkarkhi AFM, Easa AM (2010). Effect of extraction conditions on yield and degree of esterification of durian rind pectin: An experimental design. Food Bioprod. Process..

[43] Gharibzahedi SMT, Smith B, Guo Y (2019). Pectin extraction from common fig skin by different methods: The physicochemical, rheological, functional, and structural evaluations. Int. J. Biol. Macromol..

[44] Ngouémazong ED, Christiaens S, Shpigelman A, Van Loey A, Hendrickx M (2015). The emulsifying and emulsion-stabilizing properties of pectin: A review. Compr. Rev. Food Sci. F.

[45] Lewandowska K, Dąbrowska A, Kaczmarek H (2012). Rheological properties of pectin, poly(vinyl alcohol) and their blends in aqueous solutions. E-Polymers.

[46] Chen C, Huang X, Wang L-J, Li D, Adhikari B (2016). Effect of flaxseed gum on the rheological properties of peanut protein isolate dispersions and gels. LWT Food Sci. Technol..

[47] Wang T (2023). Influence of extraction methods on navel orange peel pectin: structural characteristics, antioxidant activity and cytoprotective capacity. Int. J. Food Sci. Technol..

[48] Sarojini P (2023). Design of V2O5 blocks decorated with garlic peel biochar nanoparticles: A sustainable catalyst for the degradation of methyl orange and its antioxidant activity. Materials.

[49] Nachimuthu S (2022). *Lawsonia inermis* mediated synthesis of ZnO/Fe_2_O_3_ nanorods for photocatalysis—Biological treatment for the enhanced effluent treatment, antibacterial and antioxidant activities. Chem. Phys. Lett..

[50] Surendran P (2021). Fluorescent carbon quantum dots from *Ananas comosus* waste peels: A promising material for NLO behaviour, antibacterial, and antioxidant activities. Inorg. Chem. Commun..

[51] Wang H, Chen J, Ren P, Zhang Y, Onayango SO (2021). Ultrasound irradiation alters the spatial structure and improves the antioxidant activity of the yellow tea polysaccharide. Ultrason. Sonochem..

